# How to manipulate droplet jetting from needle type jet dispensers

**DOI:** 10.1038/s41598-019-56198-0

**Published:** 2019-12-23

**Authors:** Thanh Huy Phung, Kye-Si Kwon

**Affiliations:** 1 Department of Electronic Materials and Devices Engineering, Soonchunhyang University, 22, Soonchunhyang-Ro, Shinchang, Asan, Chungnam 336-745 South Korea; 2 Department of Mechanical Engineering, Soonchunhyang University, 22, Soonchunhyang-Ro, Shinchang, Asan, Chungnam 336-745 South Korea

**Keywords:** Mechanical engineering, Surface patterning, Fluid dynamics

## Abstract

The needle-type inkjet dispenser has been widely used for various research and industrial purposes. The droplet jetting from the dispenser is closely related to the needle motion, which strikes against the nozzle seat. The strike of the needle on the nozzle seat often cause additional impact due to the bounce back, which may produce multiple droplets per jetting trigger. However, the needle motion is difficult to measure, and the actual behaviors have been known little. In this study, we measured the needle motion using an accelerometer and visualized jetting images to understand jetting behavior in relation to the needle motion. Then, we investigated various parameter effects on needle motion and jetting behaviors based on our proposed measurement methods. From the experimental results, we found that needle travel distance should be in the optimal range in order to produce single droplet per jetting trigger. In conclusion, we proposed an effective parameter selection method for the optimal jetting based on understanding of the jetting physics.

## Introduction

The needle-type jet dispensers have been used widely in both industry and research due to its capability of depositing relatively high viscous materials up to 1,000 cP (centipoise^)^^1,2^. Since the droplet deposition is based on non-contact jet mechanism, the precision control of stand-off distance (gap between the nozzle and printing media) is less required for uniform printing on a substrate when compared to conventional contact dispensing methods^3^. The droplet jetting from the jet dispenser systems is directly related to the motion of a needle, which hits the nozzle seat to produce droplet jetting^3,4^. Due to the relatively higher jetting force than that of piezo type inkjet printhead, the jet dispensers can print patterns using jetting materials with wider range of viscosity. To understand the jetting physics, experimental and numerical simulation studies have been conducted^5–8^. Shu *et al*.^4^ derived an analytical model in order to predict fluid velocity of dispensed liquid, and investigated the deposited droplet on the surface by changing jet parameters. Later, several models including simplified fluidics^1,7,9,10^, and complex dynamical models^5,8,11^ have been proposed. Most of the models are intended for better prediction of the liquid solution flow caused by the needle motion.

To produce desired droplets from the dispenser nozzle, the proper needle motion is essential. The needle motion is stopped and returned to stand-by status as a result of the impact on the nozzle seat. At the time of the impact, the droplet can be effectively jetted because the kinetic energy of the needle motion can be maximized. After the impact, the needle is likely to re-bounce, which can produce additional jetting. However, the collision effect on jetting has been ignored in most previous studies. As a result, even though previous theoretical studies provide valuable insights regarding the jet dispensing mechanism, the predicted jetting phenomenon might significantly differ from the actual jetting behavior of the dispenser^5,8^.

For the jetting behavior measurement of jet dispensers, high-speed cameras have been commonly used for the visualization by capturing sequential jet behavior with high frame rates^11–13^. However, there might be limitations in using high-speed camera to investigate the jet behaviors of dispensers. High-speed imaging requires the post-processing of captured large numbers of images, which is transferred from the camera to the computer. As a result, real-time analysis of jet images would be difficult, which is often required for gaining better physical insights during experiment. In addition, due to handling of large number of images, the use of high-speed camera might not be suitable for observing jet behavior over a long period of time. Note that we need to investigate the jet behavior for long period of time in order to understand the repeatability of jet droplets.

To overcome the drawbacks of using high speed camera imaging methods, drop visualization based on strobe LED has been used to investigate the drop formation from piezo inkjet heads^14,15^. In this study, we extended previous strobe LED visualization using a CCD (charge coupled device) camera with a low frame rate of 30 fps (frames per second) in order to understand the jet behavior from needle type dispensers.

However, without proper understanding of needle motion, visualized jet images might give limited physical insights on jet behavior. To the best of the authors’ knowledge, the needle motion effects on jetting have been known very little, because it is difficult to measure the needle movement during the jetting process. For better understanding of jetting physics, we propose a measurement method using an accelerometer to estimate the needle motion, by measuring the vibration caused by collision of the needle either on the nozzle seat (lower limit), or on the stopper (upper limit). The vibration measurement was synchronized to the jetting image acquisition in order to understand jet behavior in relation to needle motion. Various parameter effects on jetting behavior are investigated based on our proposed measurement methods for jet visualization and needle motion. Finally, we propose methods for selecting optimal parameters for jetting, so that jetting performance could be maximized without any printing defects.

## Results and Discussion

### Needle motion effects on jetting

The main driving force of jetting in the jet dispenser is the pressure generated from needle impact on the nozzle seat (Refer to Supplementary Document in order to understand working principle and parameters for controlling jet dispensers).

The pressure for jetting is proportional to the impact velocity of the needle, which is related to the travel distance of the needle. Therefore, in order to understand the jetting behaviors, the needle travel distance should be understood. Since the needle motion is difficult to measure during the jetting process, we propose an indirect measurement method based on the vibration signal measured from an accelerometer attached to the dispenser housing. Note that the impulsive vibration could be measured by using the accelerometer when the needle strikes against either the nozzle seat (bottom limit) or stopper (top limit). In this way, we can estimate the travel distance of the needle, without inserting any displacement sensors inside the dispenser. Refer to the material and method section for details of the experimental setup and measurement methods.

**Figure 1:**
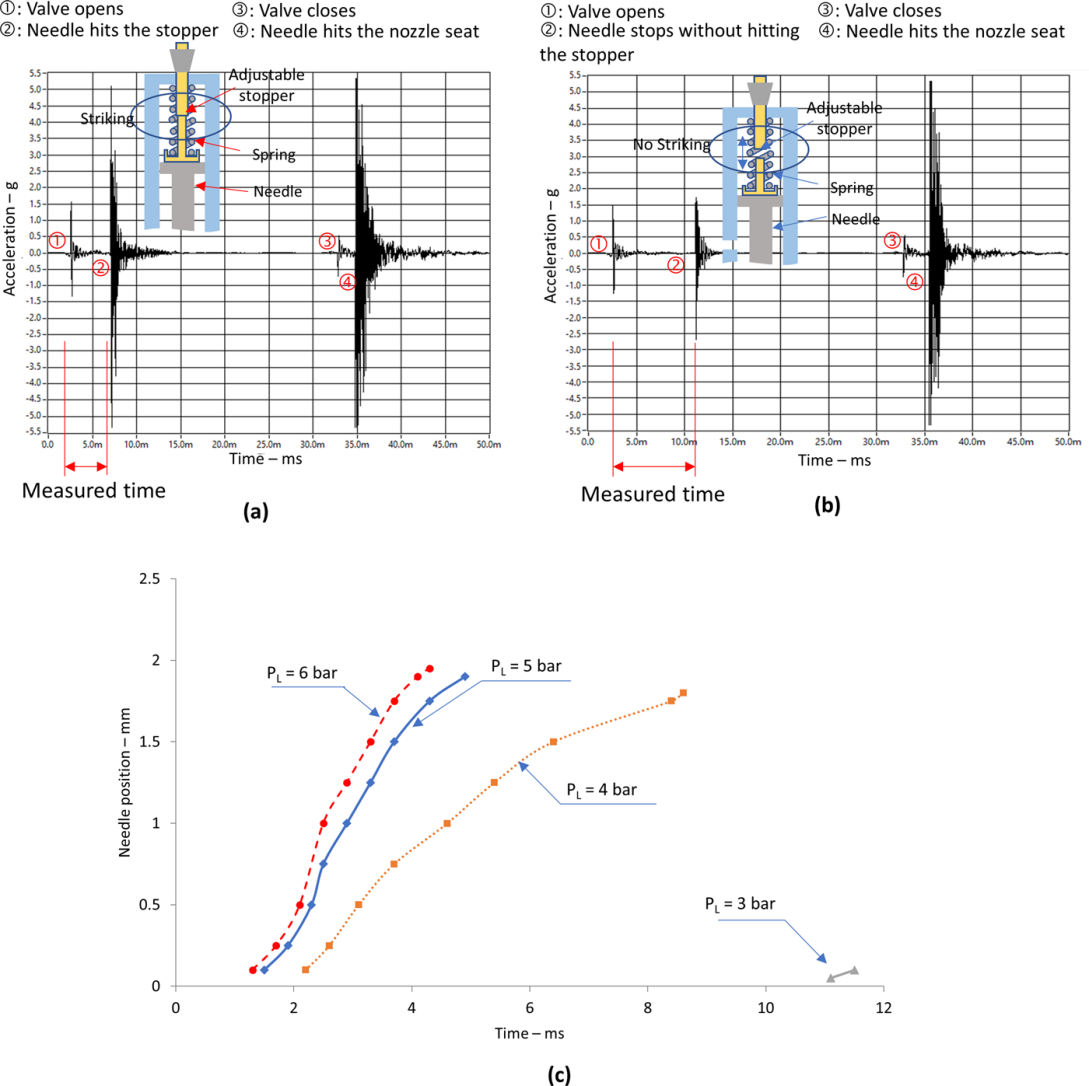
Typical vibration signal for measuring the needle motion. (**a**) Typical vibration signal when the needle hits the upper stopper; (**b**) Typical vibration signal when the needle motion stops due to the force equilibrium without hitting the stopper. (Ink supply pressure *P*_*S*_ = 0.15 bar, valve open-time, *T*_*ON*_ = 30 ms.); and (**c**) Travel distance of needle with respect to lift pressure, *P*_*L*_ and valve open-time, *T*_*ON*_.

Figure 1 shows typical vibration signals measured during whole jetting cycle when ink supply pressure was set to *P*_*S*_ = 0.15 bar. Here, we intentionally set the valve open-time, *T*_*ON*_, to be sufficiently long (*T*_*ON*_ = 30 ms) in order to investigate four steps of the needle motion as:

1. Needle up movement
  When the valve opens, the needle is lifted by the lift pressure, *P*_*L*_. As shown in initial impulsive vibration in Fig. 1(a,b) (marked as ①), it took (2–3) ms for the mechanical valve to open and compressed air to start to lift the needle. During the upward needle movement, we observed vibration with small amplitude.
2. Stop of needle lift motion
  In the case of using sufficiently long *T*_*ON*_, the needle lift motion stops due to either the arrival at the stopper position (Fig. 1(a)), or force equilibrium (Fig. 1(b)). Note that if the forces from the compressed spring and pressurized air are balanced, the needle motion could stop. In both cases, the needle maintained the lifted status for a while, until the valve was closed at *T*_*ON*_. We observed impulsive vibration signals for both stopping cases of the needle. Here, we can clearly differentiate the two stopping cases since the amplitude of the impulse vibration signals differ from the two different cases of hitting the limit stopper (marked as ② in Fig. 1(a)) and the force equilibrium conditions (marked as ② in Fig. 1(b)).
  On the other hand, in the case of using shorter *T*_*ON*_ than that of the two stopping cases, the motion can reverse its direction without arrival at the stopper or force equilibrium status when the valve is closed at *T*_*ON*_.
3. Needle down movement
  When the valve for lift pressure closes at *T*_*ON*_ (marked as 3 in Fig. 1(a,b)), the needle goes down while gaining speed via energy conversion of the potential energy (spring and gravity) to kinetic energy (needle speed). During the movement, vibration with very small amplitude can be observed.
4. Striking of the needle against the nozzle seat to produce jetting

The timing of the impact can be easily understood, because the vibration amplitude becomes largest (marked as ④ in Fig. 1(a,b)).

As shown in Fig. 1(a,b), we can understand the timing of sudden needle motion (acceleration) from the vibration signal. However, the vibration signal does not have information regarding needle travel distance. In order to estimate the needle travel distance, we propose the use of the adjustable stopper. By using the pre-determined location of the upper stopper, we can understand the travel distance of the needle from the impact vibration signal. In addition, the required travel time for the needle to reach a target distance can be measured, which is useful information for jetting parameter optimization. Note that, the valve open-time and needle travel distance have, to a certain extent, a proportional relationship. The lifted status can last until the restoration force of the spring becomes dominant by closing the valve at *T*_*ON*_.

Note that valve open-time, *T*_*ON*_, longer than the required travel time for the needle to reach maximum travel distance (location of force equilibrium status) is not efficient. To understand the relationship between travel distance (force equilibrium status) and various parameters, we plotted our experimental results as shown in Fig. 1(c). To obtain the experimental results shown in Fig. 1(c), we intentionally adjusted the stopper distance from the non-hitting location, down to the threshold status where the needle starts to hit the stopper. In this way, we could understand the needle travel distance by identifying the impulsive vibration. Here, the ink supply pressure, *P*_*S*_, was set to 0.15 bar, so that ink dripping due to the pressure could be minimized (Refer to Supplementary Document S3).

The use of proper lift pressure, *P*_*L*_, was important. For example, Fig. 1(c) shows that if the needle lift pressure is as low as 3 bar, the needle cannot be lifted properly. If the lift pressure is increased to more than 4 bar, the needle travel distance is proportionally increased with respect to *T*_*ON*_ up to 2 mm. When the needle travel distance approaches 2 mm, we observe non-linear behavior, possibly due to the non-linearity of the spring. In our study, we limited our investigation to 2 mm of needle travel distance, taking into consideration the linearity. On the other hand, if the lift air pressure uses more than 6 bar, the air in the tubing and the supply system is likely to be compressed, rather than acting as lift force on the needle. As a result, the stroke increase effects become smaller, when the lift pressure increases more than 6 bar as shown in Fig. 1(c). Considering the results, we used the lifting pressure ranging from 4 to 5 bar in our experiments. Once lift pressure, *P*_*L*_, is determined, the valve open-time, *T*_*ON*_, can be adjusted to achieve the target travel distance of the needle, which will be discussed in detail later.

### Typical drop formation of non-contact jet dispenser

**Figure 2:**
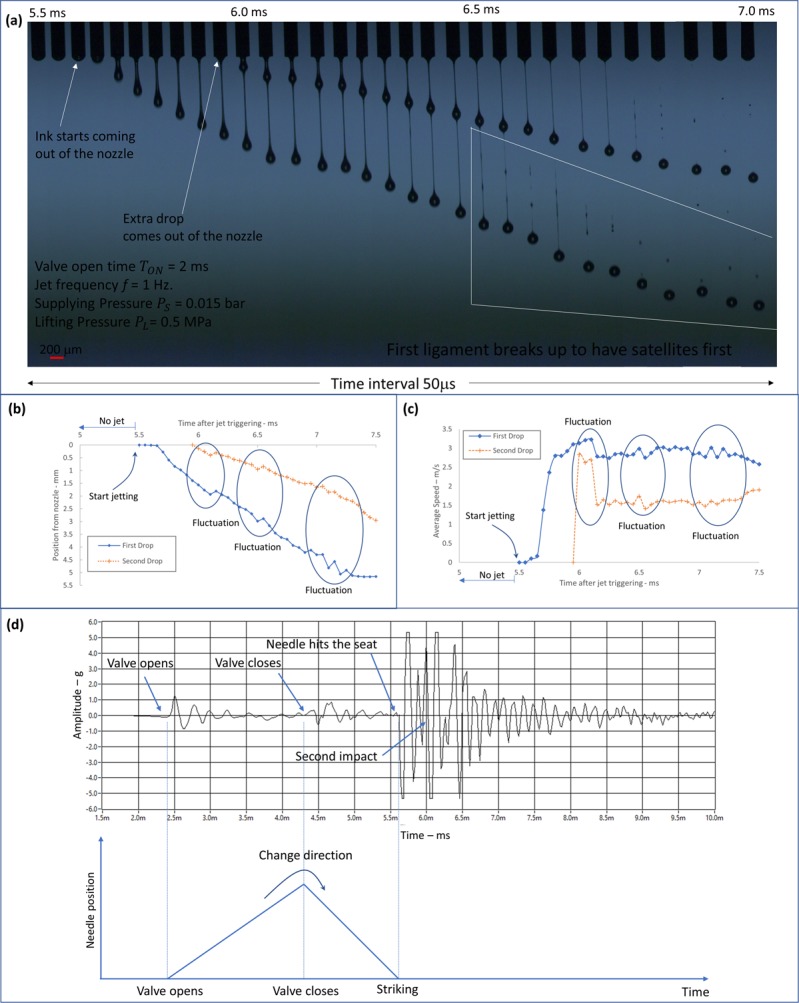
Jetting image and its analysis when *T*_*ON*_ = 2 ms is used with *P*_*L*_ = 5 bar and *P*_*S*_ = 0.15 bar. (**a**) Sequential images; (**b**) Drop formation curve; (**c**) Jetting speed curve and (see Supporting information, Movie 1); and (**d**) Vibration signal in comparison with sequential images.

**Figure 3:**
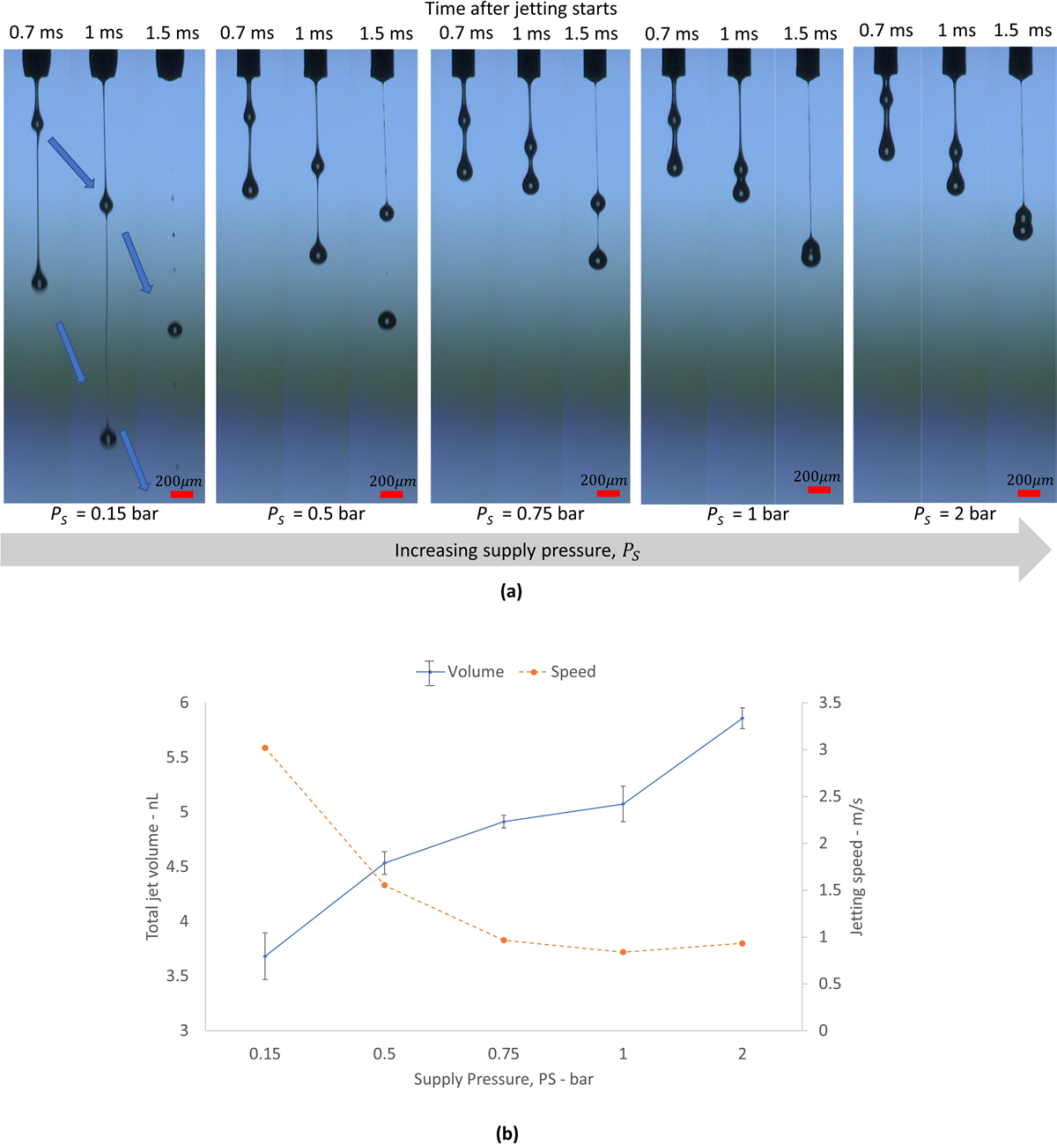
Effect of supply pressure on jetting (other jetting conditions: *P*_*L*_ = 5 bar, *T*_*ON*_ = 2 ms, and *f* = 1 Hz). (**a**) Droplet images according to supply pressure, *P*_*L*_. (**b**) Jetting speed and droplet volume plots with respect to supply pressure.

Figure 2 shows the typical jetting behavior of the needle type dispenser. For the experiment, the lift pressure, *P*_*L*_, jetting frequency, *f*, and open-time, *T*_*ON*_, were set to 5 bar, 1 Hz, and 2 ms, respectively. The needle travel distance was estimated to be about 0.25 mm by observing impulsive vibration of hitting the stopper. Here, the location of stopper was adjusted from non-hitting condition to hitting location to understand the needle travel distance. Then, the stopper position was positioned to 5 mm above the hitting location, so that the needle would not reach the stopper during the jetting process. To obtain the sequential images shown in Fig. 3(a), the trigger delay of strobe LED lights, t_d_, was increased from 5.5 to 7.0 ms with the time interval of 50 μs. Figure 2(a) shows that the initial jetting was observed around 5.6 ms after applying the jetting trigger signal. Prior to the initial material extrusion from the nozzle, the meniscus was pulled up slightly by the negative pressure caused by the lift of the needle. From the sequential images in Fig. 2(a), we observed long ligament in droplets, with the second droplet appearing at 6 ms. Note that the second droplet was formed prior to breaking up of the first droplet from the nozzle. Each droplet shows a long ligament, and the long ligaments are eventually merged with each droplet. The volume of the first and second droplets are measured to be 2.4 and 1.5 nL, respectively. Note that the vision measurement is not based on tracking a specific droplet, and each sequential image is taken from different droplets generated at the jetting frequency of 1 Hz. Due to the effects of the significant impulse vibration, we observed slight droplet location variation in the visualized images, which is not related to drop formation.

Figure 2(b) shows the drop formation curve, which plots the locations of the droplets with respect to time. The estimated jetting speeds of the main and second droplets are as low as (2–3) m/s, as shown in Fig. 2(c). In the general case of the conventional inkjet, a low jetting speed of (2–3) m/s is not likely to produce a second droplet (satellites). However, the needle type dispenser has a different mechanism to produce the second droplet and few literatures have discussed the mechanism. Note that the additional second droplet should be avoided because it could be deposited on undesired locations (see Supporting Information, Movie 2).

For better understanding of the jetting mechanism, we measured the vibration signal as shown in Fig. 2(d) and it was compared with the jetting sequential images. Here, the valve open-time, *T*_*ON*_, is set to as short as 2 ms, so that the needle could be lifted up about 0.25 mm during the valve open-time, before moving down, as illustrated in Fig. 2(d). The relationship between the needle travel distance and *T*_*ON*_ has been discussed in Fig. 1(c). Note that the impulse vibration related to the valve opening occurs at 2.4 ms, which means that the mechanical valve could take a few milliseconds to open. Then, we can observe another impulsive vibration at 5.7 ms, which is related to the impact of the needle on the nozzle seat. Note that the falling time of the needle 1.3 ms is slightly shorter than the rising time 2 ms, as illustrated in Fig. 2(d). As a result of the impact, we observed initial jetting at 5.7 ms, from the jetting images shown in Fig. 2(a). We can also observe the additional impulse vibration at 6.0 ms, which is related to the second impact of the needle. From comparison of the jetting images with the vibration signal, we can clearly understand that the second impact produces the second droplet. The additional impact of the needle against nozzle seat is caused by needle bouncing back from the first impact. As discussed in this section, the jetting behavior can be well predicted and explained by measuring vibration signal. Note that the jetting behavior can be controlled by dispenser parameters. The parameter effects on jetting will be discussed in next section.

### Jetting parameter effects on drop formation

#### Supply pressure *P*_*S*_

In order to supply ink to the dispenser chamber, air pressure (ink supply pressure), *P*_*S*_ is applied to the syringe barrel, where the ink is stored. In this section, we will discuss the influences of the supply pressure, *P*_*S*_ on the drop formation. Note that *P*_*S*_ is related to dripping of ink from nozzle rather than jetting in case when the excessive *Ps* is applied (Refer to Supplementary Document S3).

To understand the supply pressure effects on drop formation, we compared the jetting images with respect to the supply pressure as shown in Fig. 3. Interestingly, we found that the jetting speed of the first drop is inversely proportional to the ink supply pressure. Note that the closer droplet location to the nozzle means the shorter travel distance of the droplet, which means lower jetting speed since we compared the images taken at the same time from the jetting starting time. Here, the jetting starting time means the time when the jetting appears from the nozzle tip. The experimental results indicate that high pressure could counteract the needle movement when the needle moves down. Thus, it could slow down the needle speed before its impact with the nozzle seat for jetting. For better understanding, Fig. 3(b) plots the drop speed and volume with respect to the supply pressure. The figure shows that the jetting speed of the droplet reduces with increase of supply pressure while the drop volume increases (by supplying more ink to the nozzle). Here, it is obvious results that higher supply pressure could provide more material in the dispenser chamber. Note that experimental results in Fig. 3(b) are in contrast to the case of the conventional inkjet, where the higher jetting speed normally results in larger volume.

The supply pressure should not exceed 2.5 bar because of undesirable dripping on the nozzle, which causes non-jetting conditions due to the ink wetting on the nozzle tip. As a result, low supply pressure is recommended, as long as it can supply ink to the dispenser chamber.

If the first drop speed is slow enough, for example, in the cases of supply pressure more than 1 bar as shown in Fig. 3(a), the second drop could catch up with first droplet and be merged into single droplet. Single droplet jetting per one trigger signal is preferable since printing defects can be avoided (see Supporting Information, Movie 2). Next section, we will discuss how we can make single droplet jetting without producing second droplet.

#### Jetting behavior according to the needle travel distance

**Figure 4:**
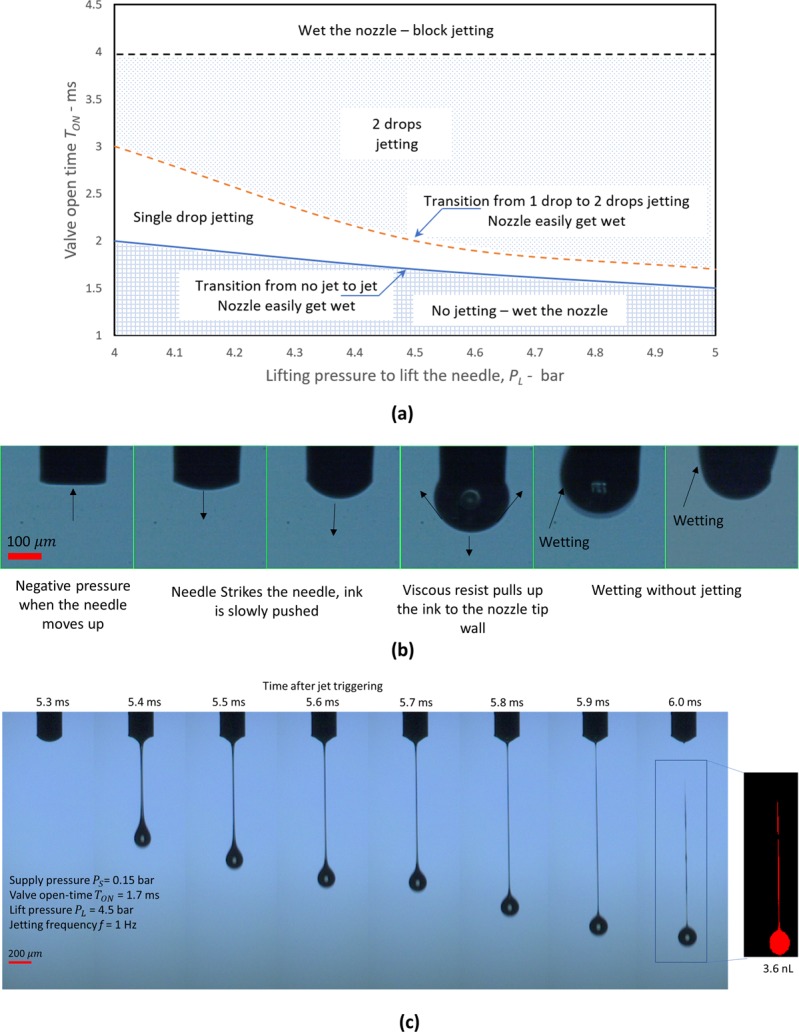
Jetting behavior according to needle motion. (**a**) Effect of parameters on jetting; (**b**) Wetting on the nozzle tip when the lift distance is about 0.08 mm. The parameters for jetting are as follow: Valve open-time *T*_*ON*_ = 1.5 ms, needle lift pressure *P*_*L*_ = 4.5 bar, supply pressure *P*_*S*_ = 0.15 bar, and jetting frequency *f* = 1 Hz; and (**c**) Typical jetting sequential images using parameters in the range of single droplet. The parameters for jetting are as follow: *T*_*ON*_ = 1.7 ms, *P*_*L*_ = 4.5 bar, *P*_*S*_ = 0.15 bar, and *f* = 1 Hz.

Needle travel distance is one of the critical parameters to produce jetting, since it is directly related to speed of needle before impact on the needle seat. To control the needle motion, two parameters are commonly used: needle lift pressure, *P*_*L*_, and valve open-time, *T*_*ON*_. To understand these parameter effects on jetting, we plotted the jettable range of parameters based on our observation, as shown in Fig. 4(a). Here, we used fixed parameters of supply pressure *P*_*S*_ = 0.15 bar and jetting frequency = 1 Hz. Note that we consider the lift pressure from 4 to 5 bar, since this pressure range is effective to lift the needle, as discussed in Fig. 1(c).

It is obvious that the shorter valve open-time, *T*_*ON*_, and smaller lift pressure, *P*_*L*_, could result in shorter lift distance of the needle or vice versa as shown in Fig. 1(c). The travel distance of the needle should be in the optimal range for proper jetting. If the needle lift distance is too short, the jetting pressure caused by needle impact is too low to cause jetting. In such a case, the nozzle tip is often wetted, as shown in Fig. 4(b). In the case of using our model fluids, the lift distance to produce proper jetting should be more than 0.1 mm. However, a longer travel distance of more than 0.2 mm often caused an undesired second droplet, due to the additional impact from bounce back of the needle. Therefore, it is important to select parameters so that a single droplet per jetting trigger signal could be produced. For this purpose, we can select parameters for jetting from the single drop jetting region shown in Fig. 4(a), which corresponds to needle travel distance of (0.1~0.2) mm.

Figure 4(c) shows typical single droplet jetting image when the parameters are selected so that travel distance of needle could be about 0.17 mm, which is shorter than the two droplets cases shown in Fig. 2(a). Here, the valve open-time *T*_*ON*_ of 1.7 ms and needle lift pressure *P*_*L*_ of 4.5 bar were used.

**Figure 5:**
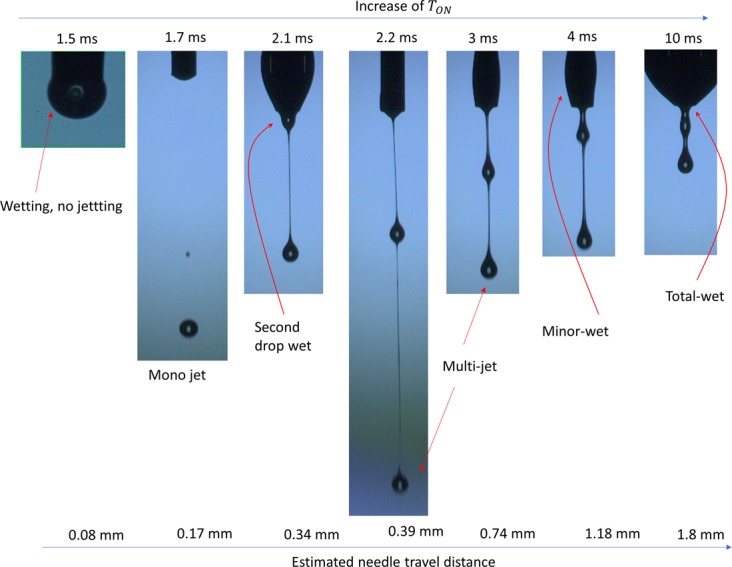
Jetting images with respect to *T*_*ON*_. All images are taken at 1 ms after ink starts coming out of the nozzle tip. The parameters for jetting are as follow: *P*_*S*_ = 0.15 bar, *P*_*L*_ = 4.5 bar, and *f* = 1 Hz, respectively. (Refer to Supporting information, Movie 3).

Even though both the needle lift pressure and the valve open-time could be used to obtain the proper needle lift distance, we recommend that the valve open-time should be the main control parameter, because of its easy controllability via printing software. For better understanding of the *T*_*ON*_ effects on jetting, Fig. 5 compares jetting images with different *T*_*ON*_. For the experiment, we set the lift pressure, supply pressure, and jet frequency as *P*_*L*_ = 4.5 bar, *P*_*S*_ = 0.15 bar, and *f* = 1 Hz, respectively. Figures 4(a) and 5 indicate that the valve open-time, *T*_*ON*_ should be in the range from 1.7 to 2.0 ms, in order to obtain proper jetting without undesirable second droplets. If *T*_*ON*_ is increased to more than 2.0 ms (above the dashed line in Fig. 4(a)), an undesirable second drop appears. When the *T*_*ON*_ increases further to more than 3 ms, the jetting speed could reduce, and wetting on the nozzle becomes dominant. During the valve open-time, the supply pressure continuously pushes the material directly to the nozzle tip, before the needle strikes on the nozzle seat for jetting. However, too much material supply could have adverse effects, causing wetting around the nozzle tip. As a result of wetting, the surface tension of wetted ink attached to the nozzle tip could significantly reduce the jet speed. If *T*_*ON*_ is increased up to 10 ms, the wetting on the nozzle surface became more serious, and could result in non-jetting status as shown in Fig. 5.

In practice, the use of the shortest possible *T*_*ON*_ is always recommended, because it can potentially increase the allowable jetting frequency. For this purpose, the lift pressure should be increased up to 5 bar to lift the needle effectively, and thereby *T*_*ON*_ could be shortened up to (1.5~1.8) ms, as shown in Fig. 4(a).

#### Jetting frequency effects on drop formation

**Figure 6:**
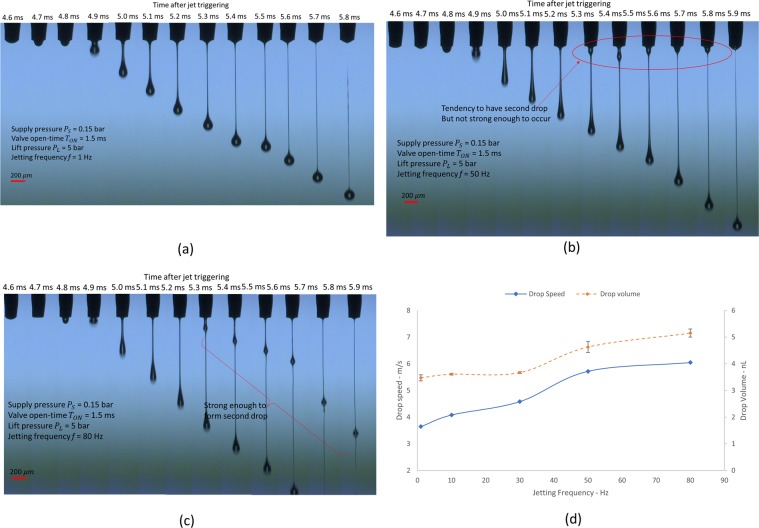
Frequency effects on jetting behavior. (**a**) Jetting frequency of *f* = 1 Hz. (**b**) Jetting frequency of *f* = 50 Hz. (**c**) Jet frequency of *f* = 80 Hz; and (**d**) Jetting behaviors according to frequencies. The parameters for jetting are as follow: *T*_*ON*_ = 1.5 ms, *P*_*L*_ = 5 bar, and *P*_*S*_ = 0.15 bar.

High jetting frequency is one of the important requirements in industrial applications, since it is related to printing performance, such as printing speed, or tact time of the dispensing process. Clearly, the maximum allowable jetting frequency is inversely proportional to the valve open-time, *T*_*ON*_, since the jetting frequency should be lower than the inverse of *T*_*ON*_. In addition, one cycle of jetting requires extra travel time for the needle to strike the nozzle seat after valve closing. Also, we might need to consider the decay time of the impulse vibration caused by the needle strike, in order for the dispenser to return to stand-by status. Based on the measured vibration shown in Fig. 2(d), one complete jetting cycle may require about 10 ms, when we used valve open-time *T*_*ON*_, of 2 ms. Considering the required time for one complete cycle, jetting frequency of lower than 100 Hz should be considered for consistent droplet jetting. However, even though we used jetting frequency of less than 100 Hz, we observed that the jetting behaviors significantly differed, according to the jetting frequency. For better understanding of the frequency effects, we compared sequential jetting images with different jetting frequency of (1, 50, and 80) Hz, as shown in Fig. 6. From the frequency sweep experiment, we can obtain plots of jetting speed and droplet volume with respect to jet frequency as shown in Fig. 6(d). The experimental results shown in Fig. 6 indicate that higher frequency could lead to higher jetting speed and higher jet amount. Interestingly, the undesired second droplet appeared when jetting frequency increased to more than 50 Hz, as shown in Fig. 6(b). When the jetting frequency becomes as high as 80 Hz, the second drop was jetted (Fig. 6(c)).

One of the possible reasons for different jetting behavior according to jet frequency is that the ink viscosity might be affected by the ink temperature, which might be increased by the energy dissipation due to frequent movement of needle (inside viscous fluid) as well as the frequent impact of the needle on the nozzle seat. In addition, in the case of shear thinning fluids, high shear rate due to the higher frequency of needle movement could reduce the equivalent viscosity of ink. As a result of ink viscosity reduction, the needle impact speed could be increased according to the increase of jetting frequency, which results in faster and higher volume jetting.

**Figure 7:**
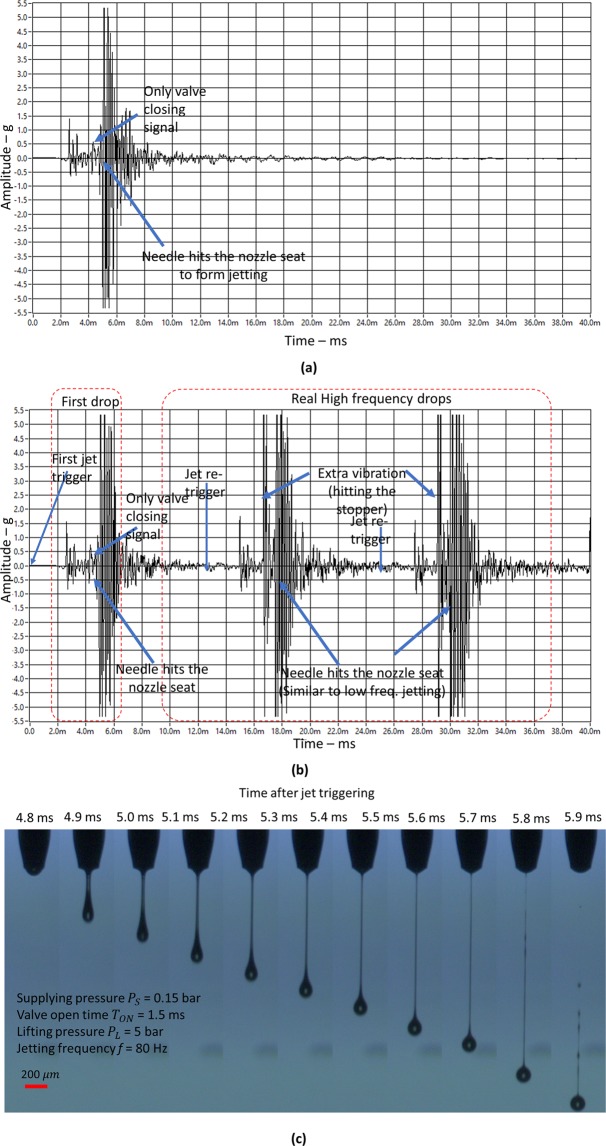
Jetting according to jetting frequency. (**a**) Vibration signal of 1 Hz jetting; (**b**) Vibration signal of 80 Hz jetting; and (**c**) Jetting images of 80 Hz after limiting the travel distance by using the stopper. The parameters for jetting are as follow: *T*_*ON*_ = 1.5 ms, *P*_*L*_ = 5 bar, *P*_*S*_ = 0.15 bar, and travel distance limit by stopper = 0.2 mm.

To understand the needle motion behavior with respect to jetting frequency, we adjusted the stopper location to restrict the allowable lift distance to 0.2 mm. In this way, we could understand whether the needle travel distance becomes more than 0.2 mm, by monitoring the additional impulse vibration signal caused by needle hitting against the stopper. For reference and comparison, the vibration signal of 1 Hz jetting was measured, as shown in Fig. 7(a). Here, we did not observe additional impulse vibration related to collision of the needle against the upper stopper, which indicates that the needle travel distance was less than 0.2 mm. However, when we increased the jetting frequency to 80 Hz, we could observe an additional strike, which happened earlier (~4 ms after triggering, right before the valve closes) than the strike on the nozzle seat at ~(4.8–5) ms after triggering), as shown in Fig. 7(b). This indicate that the needle travel distance exceeds 0.2 mm. However, interestingly, the vibration signal of the first droplet jetting shown in Fig. 7(b) shows exactly the same behavior of low-frequency jetting of 1 Hz. This means that the first droplet jetting has travel distance less than 0.2 mm. Then, the vibration signal changed from the second droplet jetting. This indicates that in the case of 80 Hz jetting, the first drop will have a single droplet, and then the rest of the droplets will have a second droplet.

Note that in the case of limiting the travel distance to 0.2 mm by using the stopper, we observed single droplet jetting, as shown in Fig. 7(c), even in the case of the jetting frequency of 80 Hz. This is different jetting behavior compared to the case when there is no stopper shown in Fig. 6(c). The experimental results indicate that the impact speed of the needle could be maintained similarly irrespective of the jetting frequency by limiting the needle travel distance. The experimental results confirmed that jetting behavior is closely related to the travel distance of the needle. Also, we can obtain uniform jetting performance without respect to jetting frequency by limiting needle travel distance.

## Concluding Remarks

In this study, we investigated the jetting physics of the non-contact inkjet dispenser in relation to various parameters. To understand the jetting behavior, we proposed the use of an accelerometer to estimate the needle movement inside of the dispenser. From the experimental study, we found that the needle lift distance during valve open-time plays critical roles in jetting and drop formation. Based on our experimental study, the parameter ranges can be divided into three different jetting conditions: non-jetting, single droplet jetting, and multiple droplet jetting. The parameters for producing the single droplet per jetting trigger are considered as the optimal conditions. We found that the lift distance of the needle should be in the optimal range, in order to produce a single droplet per trigger signal. For example, the needle lift distance of shorter than 0.1 mm could not produce jetting. On the other hand, if the travel distance becomes longer than 0.2 mm, we often observe unwanted second drop.

Based on our experimental observation, we present the optimal parameter selection methods by summarizing the parameter effects on droplet jetting.

1. *Ink supply pressure effects* (*P*_*S*_)
  We found that the ink supply pressure could affect drop formation, even though it may not directly affect needle travel distance. Excessive ink supply pressure was found to have adverse effects on jetting, because it could not only slow down jetting speed, but also cause undesirable dripping. For example, in the case of model fluid with viscosity of 140 cP, the ink supply pressure should be lower than 2.5 bar.
2. *Jetting parameter selection method*
  Based on our experimental study, we propose the parameter selection approach for valve open-time *T*_*ON*_ and lift pressure, *P*_*L*_, as follows. As a first step, we recommend that the lift pressure should be increased to be as high as possible. High lift pressure was effective in lifting the needle with faster speed, and thereby *T*_*ON*_ can be reduced, in order to lift the needle to the target location. However, lift pressure of more than 6 bar was not effective, since the air can be easily compressed in the supply system. After selecting the lift pressure, valve open-time, *T*_*ON*_, should be selected to control the needle travel distance. The valve open-time had a proportional relationship to the needle travel distance up to 2 mm. From the experimental results, we found that there was an optimal range of needle travel distance for proper jetting. For example, the needle travel distance of (0.1–0.2) mm could be considered as optimal range, since it could produce single droplet jetting per jetting trigger. For this purpose, in the case of using 5 bar for lift pressure, *T*_*ON*_ could be selected in the range from 1.7 to 2 ms. Note that the optimal needle travel distance might differ according to the viscosity of jetting material. For example, longer needle travel distance may be required for jetting of ink with higher viscosity. However, longer needle travel distance may cause problems of unwanted second droplet.
3. *Jetting frequency effects*

In practical printing, jetting frequency of less than 100 Hz is recommended by considering the required time for one complete cycle of jetting process. From the experimental results, we observed that jetting behavior was subject to jetting frequency. For example, when the frequency increases close to 100 Hz, needle travel distance becomes higher, which increases jetting speed with increase in jet volume. Also, the higher jetting frequency could result in unwanted additional drop jetting, due to the increase in needle travel distance. To prevent the unwanted second droplet, we have shown that a stopper for limiting needle travel distance could be effectively used.

## Materials and Methods

### System and measurement methods

**Figure 8:**
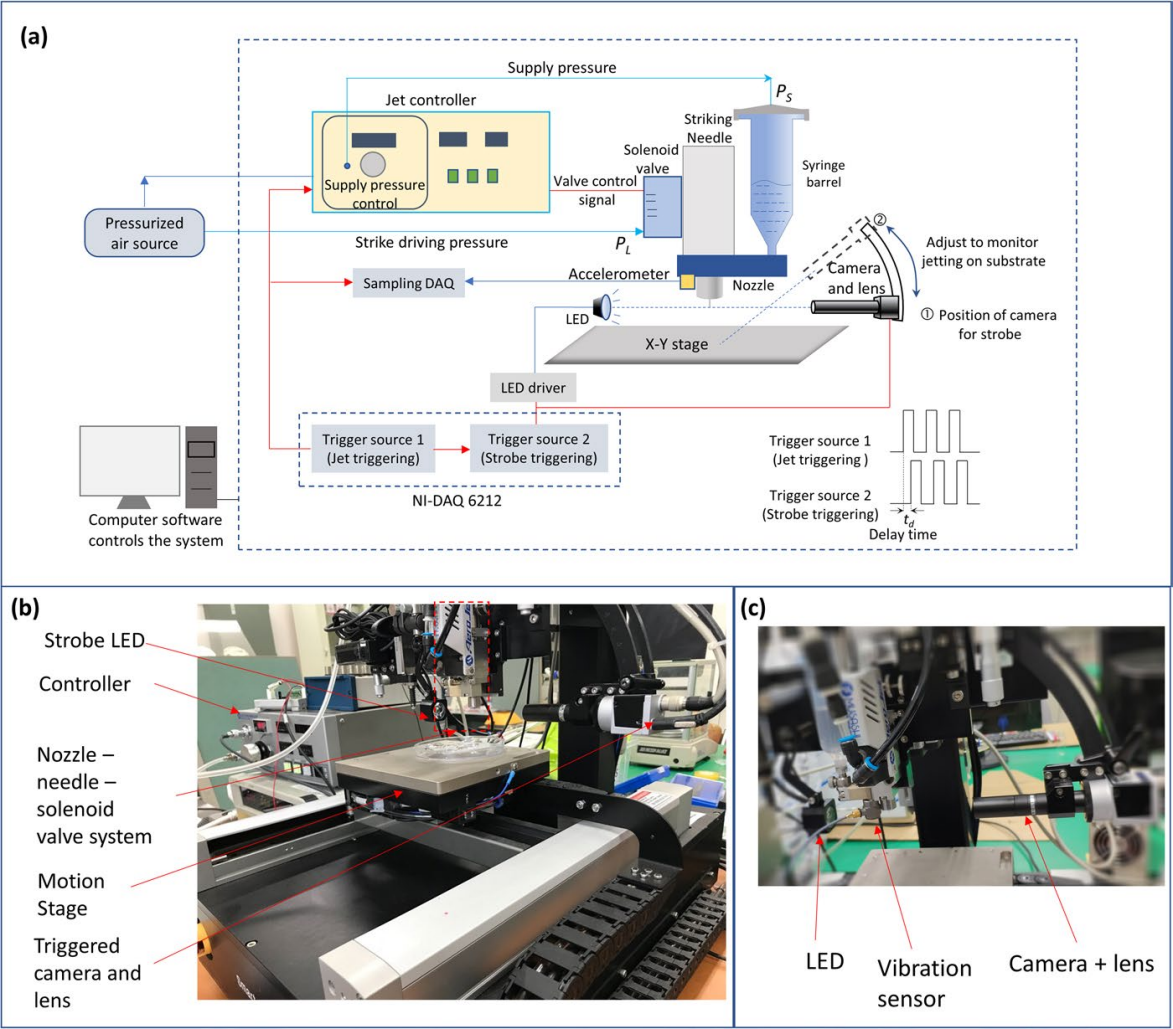
Visualization and printing system for jet-dispenser. (**a**) Schematic of jet visualization, (**b**) Photo of Experimental Setup, and (**c**) Photo of the accelerometer attached to the system.

For experimental study, we used a commercially available needle type dispenser (Aerojet dispenser, Mushashi Engineering Inc., Japan). The ink for jetting is supplied from a syringe barrel by a supply pressure, *P*_*S*_. The droplet jetting from the dispenser is controlled via a solenoid air valve. During the valve open time, *T*_*ON*_, a lift pressure, *P*_*L*_, is applied to lift the needle. When the valve close, the needle is returned back to produce droplet jetting by hitting the nozzle seat. (Refer to Supplementary Document S1). To visualize the jet behavior of the jet-dispenser system, we developed an in-house drop visualization system. In the system, on-off status of the solenoid valve for the lift pressure, *P*_*L*_, was controlled by digital signals. Here, the on-time of the digital signal corresponds to valve open-time, *T*_*ON*_, for lifting the needle. For this purpose, the digital pulse train with adjustable duty ratio (jetting trigger signal) was generated from a multi-function data acquisition device (Counter out 1, NI DAQ 6212, National Instrument, USA). The digital signals are also used to trigger another (second) digital pulse train having an adjustable trigger delay time, t_d_, as shown in Fig. 8(a). The second digital pulse train (Counter out 2, NI DAQ 6212, National Instrument, USA) was used as trigger signals for both strobe LED lighting and a CCD camera (Basler Aca1920–1500uc, Basler AG, Germany). Here, the CCD camera was equipped with lenses of 1x magnification (MML1ST65, Moritex Corp., Japan). As a result of synchronized LED lighting, the frozen jetting images at t_d_ could be acquired from the camera. Moreover, by increasing the delay time, t_d_, from the starting time to the target ending time, sequential jetting images could be obtained. Note that the CCD camera was triggered to acquire an image of a specific droplet, which is called as single strobe method in contrast to strobe LED measurement^16^. The camera trigger can acquire a specific droplet image only, and can avoid the averaged image of repeated individual droplets. In the case of using conventional strobe LED measurement, which does not use a camera trigger, the droplet image might appear blurred, if the droplet location varies time to time. Note that we often observed slight variation of droplet location during our experiment, due to the impact vibration from needle strikes.

Figure 8 shows schematic and photo of jet dispensing system for our experiment. To visualize the jetting behavior, the camera and LED light were aligned horizontally (position 1 in Fig. 8(a)). On the other hand, our experimental system has the capability of adjusting the camera viewing-angle so that the camera view could focus on the substrate during printing when the droplet placement behavior on the substrate is monitored (position ② in Fig. 8(a)). By using visualization of the droplets in flight, jetting behaviors such as jetting speed and droplet volume could be measured (Refer to Supplementary Document S2).

For better understanding of the jetting physics, it is important to measure the needle motion during jetting. However, direct measurement of the needle motion using a sensor is difficult, because it is difficult to install on the moving needle inside of the dispenser chamber. To overcome these difficulties, we proposed the indirect measurement of needle motion using a piezo-type accelerometer (Type 4513-B-001, Brüel & Kjær, Denmark) attached to the dispenser housing as shown in Fig. 8(c). To acquire vibration data from the accelerometer, data acquisition system (cDAQ 9174, NI, USA) is used, with a sample rate of 50 kHz. Here, we set the accelerometer amplifier to have the gain of 0.1022 V/g, where g is 9.81 m/s. To synchronize the vibration measurement with respect to jetting, the jetting trigger is used as a trigger signal for the acquisition of vibration data.

### Jetting materials

A mixture of distilled water (10 wt.%) and Glycerin (90 wt.%) (Duksan Pure Chemicals, S. Korea) was used as a model fluid in the experiments. The viscosity and surface tension of mixed solution measured by rheometer (Brookfield DV-III, Ametek Inc., USA) and a Du NÕuy ring analyzer set (DST30, SEO, South Korea) at 25 °C were about 140 cP and 63 mN/m, respectively.

Note that the jetting behavior from the dispenser may differ according to the jetting material. Nevertheless, we tried to generalize our study so that the results and conclusions could be easily extended to other jetting materials.

## Supplementary information

Supplementary information

Supplementary information 2

Supplementary information 3

Supplementary information 4
