# Supplementary material for: How to manipulate droplet jetting from needle type jet dispensers
Source: Sci Rep. 2019 Dec 23;9:19669. doi: 10.1038/s41598-019-56198-0 (PMC6928253; doi:10.1038/s41598-019-56198-0)
Supplement: Supplementary file 1 — Supplementary information [file 41598_2019_56198_MOESM1_ESM.docx]

**Supplementary Document**

**How to manipulate droplet jetting from needle type jet dispensers**

Thanh Huy Phung^1^, Kye-Si Kwon^2,*^

^1^*Department of Electronic Materials and Devices Engineering, Soonchunhyang University, 22, Soonchunhyang-Ro, Shinchang, Asan, Chungnam, 336-745, South Korea*

^2^*Department of Mechanical Engineering, Soonchunhyang University,
22, Soonchunhyang-Ro, Shinchang, Asan, Chungnam, 336-745, South Korea*

** Email: kskwon@sch.ac.kr*

**S1. Working principle of needle type jet dispenser for droplet jetting**

Fig. S1 shows the basic components of the dispenser. Throughout our experiment, a nozzle with inner diameter of 100 $\mu m$ and nozzle length of 5 mm was used. In order to control the needle motion, a solenoid valve is used for on-off control of pressurized air (lift pressure) to lift the needle, by overcoming the compressed spring force. Here, two parameters have been used for this purpose: the valve open-time, $T_{ON}$, and lift pressure, $P_{L}$. Note that a spring is mounted between the needle and housing to produce the restoration force of the lifted needle. The maximum travel distance (stroke) of the needle could be limited by a stopper, which could be adjusted via a micrometer. In order to supply the jet solution to the injection chamber, a supply pressure, $P_{S}$, was applied to the syringe barrel.


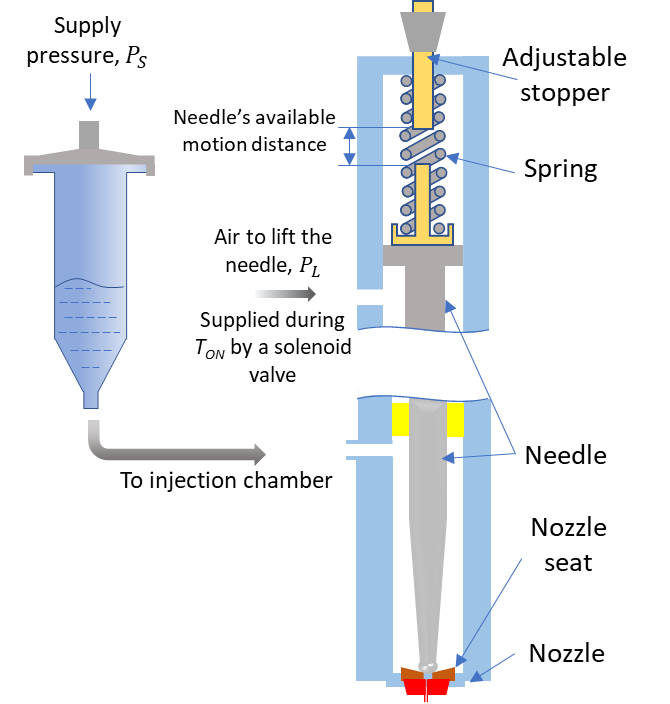


FIG S1. Basic components of the dispenser used in the experiments.

In general, the inkjet dispensing process requires several stages of needle movement as illustrated in Fig. S2:

➀ Needle resting stage:

At the beginning, the needle rests on the nozzle seat, due to the preloaded spring. The needle blocks the nozzle orifice to prevent ink from flowing from the nozzle, while the jet solution is supplied by the supply pressure, $P_{S}$.

➁ Needle lifting up stage:

The solenoid valve for controlling on-off of lift air pressure, $P_{L}$, is open, in order to lift the needle during a period of valve open-time, $T_{ON}$.

➂ Needle moving down stage:

At $T_{ON}$, the solenoid valve is closed to remove the lift pressure, $P_{L}$. The restoring force from the compressed spring returns the needle to the nozzle seat.

➃ Needle striking stage:

The moving needle gains kinetic energy (speed), before it reaches maximum speed for striking against the nozzle seat. As a result of the high-speed impact, jetting is produced.


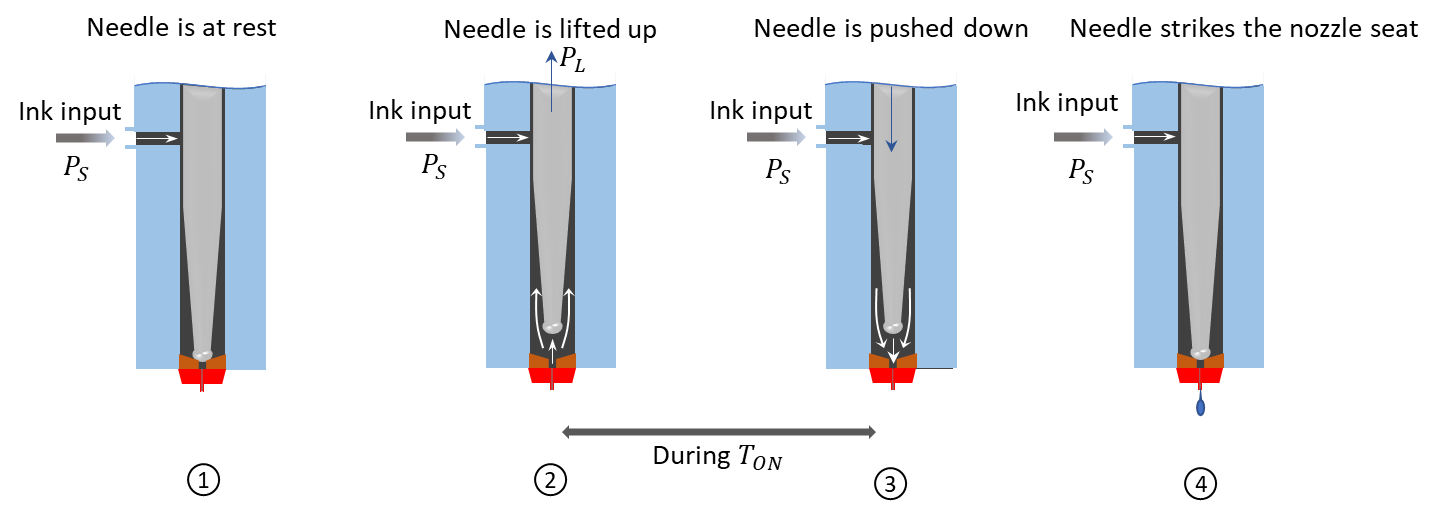


FIG S2. Needle movement stages for jetting.

In order to control needle motion, most of needle type dispensers have been using air pressure as shown in Fig.S1. However, it should be noted that other types of actuators can be used to generate needle motion for droplet jetting. For example, as an alternative method for lifting the needle, either a piezo-actuator or a magnetic actuator [S1, S2, S3, S4] could be used. Nevertheless, the basic working principles are similar to that of the needle type dispenser described in Fig. S2.

**S2. Image processing method for jetting behavior measurement.**


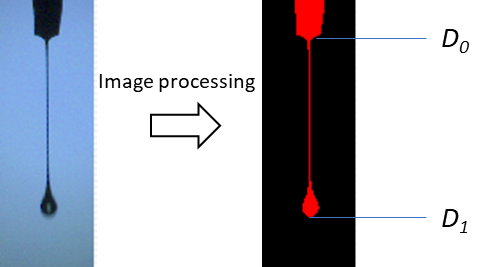


FIG S3. Binary image analysis for jetting characterization.

Figure S3 shows a typical acquired image by using strobe LED. The acquired images can be further processed for the analysis. For example, binary image analysis of the acquired images was often used to obtain droplet locations [S5, S6], as shown in Fig. S3. Then, *the average jetting speed,*$\bar{v}_{jet}$*,* can be calculated by [S5]:

$\bar{v}_{jet}\left( t_{d} \right)=\frac{D_{1}\left( t_{d} \right)-D_{0}}{t_{d}-t_{0}}$ (S1)

where $t_{0}$ and $t_{d}$is the time of first droplet appearance from the nozzle tip (*D_0_*) and the time of droplet location at *D_1_*, respectively.

From the acquired images, we calculated the volume of droplet by so-called edge detection algorithm. Inclusion of satellite droplets and long ligament (tails) of the droplet in the volume measurement has been an important issue in order to estimate droplet amount using image analysis. For this purpose, we used sufficiently long ROI (region of interest) to include satellites and the long ligament of droplets in the image analysis. The details of using ROI for droplet volume measurement has been discussed in [S6]. Figure S4 illustrate the image analysis method for volume measurement method, which creates segment of whole droplets of interest into many cylindrical elements based on the detected edges in the horizontal directions. By using the edge detection methods, volume of satellites and the tails of droplets can be included in measured droplet volume.


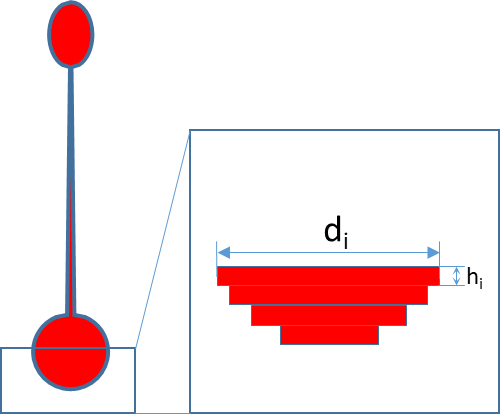


FIG S4. Volume measurement for drop ligament.

As shown in Fig. S4, the volume of the droplet volume with long ligament is calculated by:

$V=\sum\left( h_{i}\times\frac{\pi d_{i}^{2}}{4} \right)$ (S2)

where *h_i_* and *d_i_* are the edge width and height of cylindrical segment *i*, as shown in Fig. S4. The detailed algorithms could be referenced in Refs. [S5, S6]. Note that the volume measurement based on image processing may have accuracy issues, because the detected edge location can be subject to various conditions, such as imaging resolution, lens focus, and lighting condition. So, the calculated volume should be used for relative comparison only [S7]. Alternatively, a scientific electronic scale can be used for droplet mass measurement [S7]. However, for proper mass measurement, the evaporation of the deposited droplets on the scale needs to be considered [S7].

**S3. Dripping under the effect of supply pressure**

The jetting of non-contact dispenser is produced by needle impact against nozzle seat, while the solution is supplied by supply pressure, $P_{S}$. The effect of $P_{S}$ on jetting is smaller than that of the jetting pressure generated from the needle impact. As a result, the effects of $P_{S}$could be related to dripping of ink from the nozzle, rather than jetting. Note that dripping of ink due to $P_{S}$should be avoided for stable jetting. In this section, we will consider the case when only $P_{S}$ applies, excluding the needle motion effects, in order to explain the supply pressure, $P_{S}$, effects on dripping.

In order to obtain jetting rather than dripping, the inertia of fluid should be high enough to overcome the surface tension and form droplets. In such case, the Weber number, *We*, should exceed a critical Weber value, $We_{C}$ as [S8, S9]:

$We=\frac{\rho Rv^{2}}{\gamma}>We_{c}$ (S3)

where, $\rho, R, v$, and $\gamma$ are the density jetting material, nozzle inner radius, fluid speed, and material surface tension, respectively. Previous experimental works [S8–S10] indicated that a $We_{c}$ range from (1 to 4) would lead to droplet jetting. In the case of using model fluid used in our experiment (Table 1 in the main text), an average speed of fluid speed more than 1 m/s could lead to droplet jetting.

When the model fluid is pushed via $P_{S}$into the dispenser chamber, the pressure loss caused by ink flow from the syringe barrel to the dispenser chamber could be negligible, compared to that of ink flow through the nozzle tip. The pressure loss due to ink flow can be understood by the Poiseuille-Hagen equation as:

$\Delta P=\frac{8\mu Lv}{R^{2}}$ (S4)

where, *L*, *R* are the length and inner radius of the tubing (or the nozzle) that the jetting material passes through, respectively. Note that *R* is a critical parameter when considering pressure loss. If we assume that only the supply pressure, $P_{S}$, is applied for jetting, $P_{S}$ should be high enough to overcome the pressure loss and provide kinetic energy to produce more than 1 m/s of fluid flow speed through the nozzle. Based on Eq. (S4), an extremely high pressure of more than 22 bar is required to compensate the pressure loss, in the case of using a nozzle with inner diameter of 100 $\mu m$ and length of 5 mm. As a result, the supply pressure itself is not likely to produce droplet jetting since the use of air pressure more than 5 bar is not recommended for practical reasons.

The experimental results in Fig. S4 explain the ink dripping behavior when only $P_{S}$ is applied. In this experiment, the needle was lifted for a sufficiently long time in order to exclude needle strike effects. As shown in Fig. S4 (a), considerable time is required to observe drippings detached from the nozzle. Based on our experimental observation, we plotted the time required for dripping with respect to supply pressure as shown in Fig. S4 (b). Figure S4 (b) shows that high supply pressure could shorten the time required for dripping. Nevertheless, it took seconds to form drops, even in the case of high pressure of 5 bar. In general, the dripping frequency due to $P_{S}$ is very low compared to the jetting frequency used in most applications.


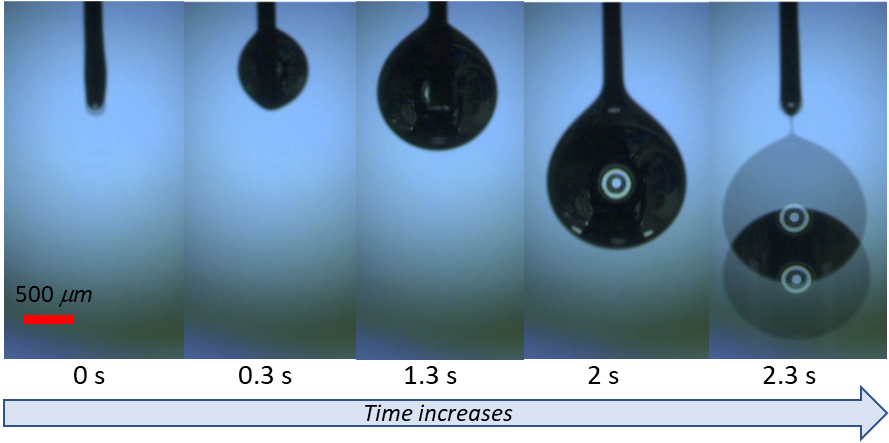


(a)


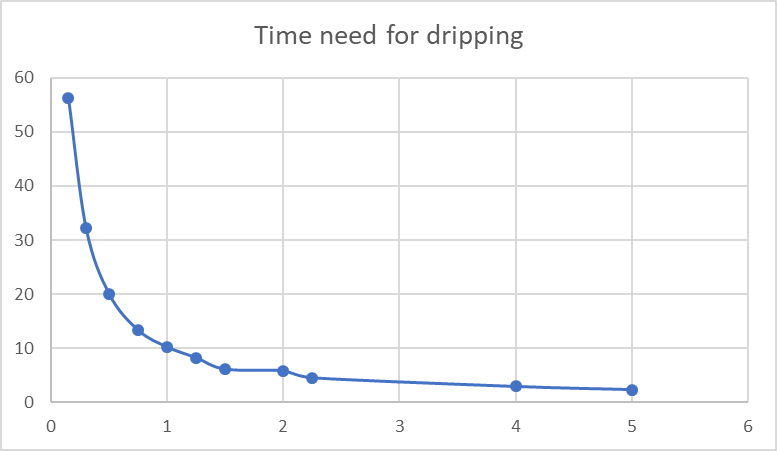


(b)

FIG S4. Dripping behavior from nozzle when only *P_S_* is applied. (a) Typical dripping images when 5 bar of *Ps* is applied. (b) Time between 2 drops with respect to supply pressure.

Equations (S3), (S4) and the experimental results in Fig. S4 offer useful information in order to gain physical insights on how much supply pressure should be applied in practice. As discussed in this section, it is clear that the supply pressure itself does not directly produce jetting. Even though the result cannot be directly used to determine the optimal supply pressure, it affects drop formation of the jetted droplet, which will be discussed in the main text.

**References**

S1. S. Lu, G. Cao, H. Zheng, D. Li, M. Shi and J. Qi. Simulation and Experiment on Droplet Formation and Separation for Needle-Type Micro-Liquid Jetting Dispenser. *Micromachines* **9,** 330 (2018). DOI: [10.3390/mi9070330](https://dx.doi.org/10.3390%2Fmi9070330)

S2. J. Li. Technology development and basic theory study of fluid dispensing – a review. In: *IEEE Proceeding of HDP’04*, 198-205 (2004). DOI: [10.1109/HPD.2004.1346698](https://doi.org/10.1109/HPD.2004.1346698)

S3. C. Zhou, J. H. Li, J. A Duan and G. L. Deng. The principle and physical models of novel jetting dispenser with giant magnetostrictive and a magnifier. *Scientific Reports* **5**, 18294 (2015). DOI: <https://doi.org/10.1038/srep18294>

S4. Q. H. Nguyen, M. K. Choi, B. Y. Yun and S. B. Choi. Design of a Novel Jetting Dispenser Featuring Piezostack and Linear Pump, J*. Intell. Material Syst. Struct*. **19,** 333-341 (2007). DOI: [https://doi.org/10.1177/1045389X07083613](https://doi.org/10.1177%2F1045389X07083613)

S5. K. S. Kwon. Experimental analysis of waveform effects on satellite and ligament behavior via in situ measurement of the drop-on-demand drop formation curve and the instantaneous jetting speed curve, J. Micromech. Microeng. **20,** 115005 (2010). DOI: <https://doi.org/10.1088/0960-1317/20/11/115005>

S6. K. S. Kwon, M. H. Jang, H. Y. Park and H. S. Ko. An inkjet vision measurement technique for high frequency jetting. *Rev. Sci. Instrum.* **85**, 065101 (2014). DOI: <https://doi.org/10.1063/1.4879824>

S7. K. S. Kwon, D. Zhang and H. S. Go. Jetting frequency and evaporation effects on the measurement accuracy of inkjet droplet amount. *Journal of imaging science and technology* **59**, 2 (2015). DOI: <https://doi.org/10.2352/J.ImagingSci.Technol.2015.59.2.020401>

S8. C. Clanet and J. C. Lasheras. Transition from dripping to jetting. *J. Fluid Mech.* **383**, 307 (1999). DOI: <https://doi.org/10.1017/s0022112098004066>

S9. W. V. Hoever, S. Gekle, J. H. Snoeijer, M. Versluis, M. P. Brenner and D. Lohse. Breakup of diminutive Rayleigh jets. *Physics of Fluids* **22**, 122003 (2010). DOI: <https://doi.org/10.1063/1.3524533>

S10. B. Derby. Inkjet printing ceramics: From drops to solid. *Journal of the European Ceramic Society* **31**, 2543–2550 (2011). DOI: <https://doi.org/10.1016/j.jeurceramsoc.2011.01.016>
